# Spontaneous bacterial peritonitis caused by *Edwardsiella tarda*: A case report

**DOI:** 10.1016/j.ijscr.2020.09.126

**Published:** 2020-09-23

**Authors:** Hirokatsu Hayashi, Yusuke Murase, Hitoya Sano, Kimitosi Nishio, Iwao Kumazawa

**Affiliations:** Department of Surgery, JA GIFU Kouseiren Ibi Kosei Hospital, 2547-4 Miwa, Ibigawa-cho, Ibi-District, Gifu-Prefecture, 501-0696, Japan

**Keywords:** SBP, spontaneous bacterial peritonitis, CT, computed tomography, MOF, multiple organ failure, *Edwardsiella tarda*, Spontaneous bacterial peritonitis, Cirrhosis

## Abstract

**Introduction:**

*Edwardsiella tarda* is a member of the *Enterobacteriaceae* family of gram-negative bacilli isolated from animals. Gastroenteritis is the most common manifestation of *E. tarda* infection. However, extraintestinal infections can occur in immunocompromised hosts as well as patients with hepatobiliary disease, malignancy, and/or diabetes mellites. The prognosis of sepsis caused by *E. tarda* is extremely poor, with a mortality rate of 38%. Here we report the occurrence of spontaneous bacterial peritonitis associated with *E. tarda* infection.

**Presentation of case:**

An 87-year-old man with Child–Pugh A cirrhosis secondary to hepatitis C virus infection presented with diarrhea and sudden-onset pain in the abdomen. On arrival, guarding and rebound tenderness were observed over the entire abdomen. Computed tomography revealed circumferential thickening of the cecum and a small volume of ascites in the pelvic cavity. A diagnosis of peritonitis was made, and surgery was performed. Surgical findings included redness and thickening of the cecal wall and purulent ascites without intestinal contents. Peritoneal fluid culture revealed *E. tarda* as the sole pathogen. The abdominal cavity was washed, a drain was placed in the pelvic cavity, and postoperative intravenous antibiotic therapy was initiated. The postoperative course was uneventful.

**Discussion:**

*E. tarda* infection in the presence of an underlying disease such as hepatobiliary disease, malignancy, and/or diabetes mellitus has a poor prognosis.

**Conclusion:**

Although *E. tarda* infection is extremely rare, it is a life-threatening illness that can cause intestinal and extraintestinal infections. If necessary, early surgical intervention should be considered for cases of extraintestinal infection.

## Highlights

• Case of spontaneous bacterial peritonitis associated with *E. tarda* infection.
• The patient had Child–Pugh A cirrhosis secondary to hepatitis C virus infection.
• Surgical findings included redness and thickening of the cecal wall.
• Purulent ascites without intestinal contents was observed.
• *E. tarda* infection has a poor prognosis in patients with underlying disease.

## 1 Introduction

*Edwardsiella tarda* is a member of the *Enterobacteriaceae* family of gram-negative bacilli isolated from animals, including fish, amphibians, reptiles, and birds [1]. Gastroenteritis is the most common manifestation [1]. However, extraintestinal infections, including soft tissue infection, sepsis, hepatobiliary infection, intra-abdominal abscess, wound infection, meningitis, osteomyelitis, endocarditis, tubo-ovarian abscess, empyema, and salpingitis, can occur in immunocompromised hosts as well as patients with hepatobiliary disease, malignancy, and/or diabetes mellites [[2], [3], [4], [5], [6], [7], [8], [9], [10], [11], [12], [13], [14]]. The prognosis of sepsis caused by *E. tarda* is extremely poor, with a mortality rate of 38% [2]. Here we report the occurrence of spontaneous bacterial peritonitis (SBP) associated with *E. tarda* infection in an 87-year-old man with Child–Pugh A cirrhosis secondary to hepatitis C virus infection. This work has been reported in line with the SCARE criteria [15].

## 2 Presentation of case

**Fig. 1:**
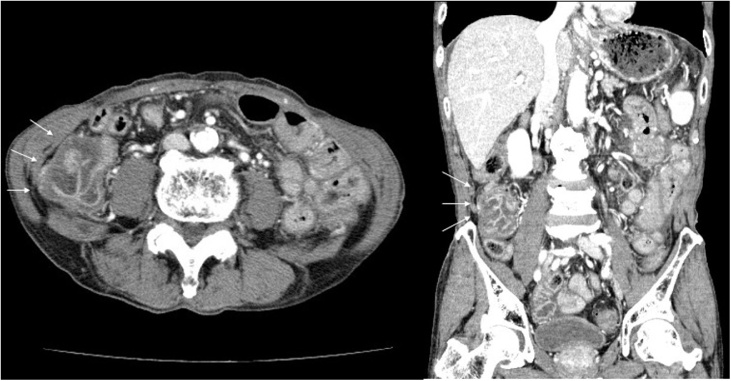
Contrast-enhanced computed tomography findings for a patient with spontaneous bacterial peritonitis caused by *Edwardsiella tarda*. The images show circumferential thickening of the cecum (arrows).

An 87-year-old man with Child–Pugh A cirrhosis secondary to hepatitis C virus infection presented with diarrhea and sudden-onset pain in the lower abdomen that gradually increased in severity. On arrival, he was conscious and alert, with a blood pressure of 139/62 mm Hg, heart rate of 98 beats/min, temperature of 39.3 °C, and a peripheral oxygen saturation of 97% at ambient air. There were no cardiovascular or respiratory abnormalities. Guarding and rebound tenderness were observed over the entire abdomen, particularly the lower quadrant. Laboratory tests revealed the following: white blood cells, 4500/μL with left deviation (neutrophils, 94.7%); C-reactive protein, 0.16 mg/dL; hemoglobin, 11.6 g/dL; platelet count, 5.2 × 10^4^/μL; prothrombin time, 11.7 s; international normalized ratio, 1.04; total bilirubin, 0.8 mg/dL; albumin, 3.9 g/dL; aspartate transaminase, 51 IU/L; alanine transaminase, 36 IU/L; and serum creatinine, 0.81 mg/dL. Computed tomography (CT) revealed circumferential thickening of the cecum and a small volume of ascites in the pelvic cavity (Fig. 1). A diagnosis of peritonitis was made, and surgery was performed to identify the cause. Surgical findings included redness and thickening of the cecal wall and purulent ascites without intestinal contents. The abdominal cavity was washed, a drain was placed in the pelvic cavity, and postoperative intravenous antibiotic therapy was initiated. The postoperative course was uneventful. Three days after surgery, peritoneal fluid culture revealed *E. tarda* as the sole pathogen. The final diagnosis was SBP associated with gastroenteritis caused by *E. tarda*. The patient was discharged 14 days after the surgery.

## 3 Discussion

SBP is defined as an ascitic fluid infection without an evident intra-abdominal, surgically treatable source that occurs in patients with decompensated cirrhosis and ascites. The clinical diagnosis is based on paracentesis showing a polymorphonuclear leukocyte count of ≥250/mm^3^ in ascitic fluid and a positive ascitic culture. *Escherichia coli* and *Klebsiella pneumonia*, which are isolated in 72% cases, are the main causative bacteria [16]. Bacterial translocation is considered the etiology of SBP. Patients with cirrhosis exhibit a predisposition to intestinal bacterial overgrowth, intestinal dysmotility, and increased intestinal permeability, all of which lead to increased bacterial translocation [[17], [18], [19]]. In order to prevent complications and improve survival, empirical antibiotic treatment must be initiated immediately after the diagnosis is established.

The colonization rate of *E. tarda* in the stool of healthy individuals is reportedly 0.007% [20]. Contact with animals such as fish, amphibians, reptiles, and birds and consumption of contaminated foods such as sushi, raw fish, and other seafood are considered risk factors for *E. tarda* infection. Gastroenteritis is the most common manifestation that spontaneously resolves without antibiotics [1]. However, as mentioned earlier, extraintestinal infections can occur in certain susceptible individuals [[2], [3], [4], [5], [6], [7], [8], [9], [10], [11], [12], [13], [14]]. Although *E. tarda* is sensitive to antibiotics with activity against gram-negative bacilli, the prognosis of sepsis caused by this organism is extremely poor [2].

In the present case, the patient had cirrhosis secondary to hepatitis C virus infection. Because gastroenteritis was recognized as a prodromal symptom and *E. tarda* was solely detected in ascites culture, a final diagnosis of SBP caused by *E. tarda* was established. We suspected that *E. tarda* caused the gastroenteritis, which then progressed to peritonitis. Although paracentesis to confirm the characteristics of the ascites was considered, puncture would have been difficult because of the small fluid volume. Eventually, laparotomy was performed to find the cause, and this facilitated rapid surgical drainage. The postoperative course was favorable because of the prompt surgical drainage and appropriate antibiotic therapy.

We also conducted a literature search and identified a total of 14 cases, including the present case, of *E. tarda* infection with a surgically treatable source of extraintestinal complications in Japan [[2], [3], [4], [5], [6], [7], [8], [9], [10], [11], [12], [13], [14]] (Table 1). It was found that most patients had a significant underlying disease, with the most common ones being hepatobiliary disease (cirrhosis, alcoholic liver injury, common bile duct stone and cholecystectomy; 7/14 [50%]), malignancy (gastric cancer and appendiceal cancer; 3/14 [21%]), and diabetes mellitus (2/14 [14%]). Ten patients, including the present patient, required surgical treatment, and all of them survived. On the other hand, four of six patients with necrotizing fasciitis could not undergo surgical treatment and died from exacerbation of sepsis that resulted in septic shock and multiple organ failure (MOF) in a short period of time and. From the four patients who died, three had hepatobiliary disease such as cirrhosis and alcoholic liver injury. In cases of liver diseases, impaired clearance of bacteria due to hypofunction of the reticuloendothelial system and decreased detoxification due to arteriovenous shunting are considered to lead to exacerbation of sepsis and, subsequently, septic shock, disseminated intravascular coagulation, and MOF [2].

**Table 1:** Cases of *Edwardsiella tarda* infection with a surgically treatable source of extraintestional complications in Japan.

No.	Author	Case	Underlying illness	Prodrome	Extraintestinal infection	Treatment	Outcome
1	Matsushima [2]	67/M	Cirrhosis	Diarrhea	Necrotizing fasciitis	(–)	Dead
2	Tamura [3]	71/M	Alcoholic liver injury	Diarrhea	Necrotizing fasciitis	(–)	Dead
3	Fujimoto [4]	75/M	Cirrhosis	(–)	Necrotizing fasciitis	(–)	Dead
4	Sekine [5]	83/F	Distal gastrectomy for gastric ulcer	(–)	Necrotizing fasciitis	(–)	Dead
5	Sugita [6]	55/M	Alcoholic liver injury	(–)	Necrotizing fasciitis	Debridement	Alive
6	Hara [7]	49/M	(–)	(–)	Necrotizing fasciitis	(–)	Alive
7	Tokushige [8]	54/F	Genital chlamydia Graves' disease	(–)	Tubo-ovarian abscess	Salpingo-oophorectomy	Alive
8	Kobayashi [9]	83/F	Appendiceal cancer Chronic renal failure Diabetes mellites	(–)	Tubo-ovarian abscess	Salpingo-oophorectomy	Alive
9	Anno [10]	76/M	Chronic subdural hematoma Common bile duct stone	(–)	Infectious subdural hematoma	Drainage	Alive
10	Ota [11]	70/F	Autoimmune hemolytic anemia Early gastric cancer	(–)	Liver abscess	Drainage	Alive
11	Ohara [12]	85/F	Diabetes mellites	Diarrhea	Liver abscess	Drainage	Alive
12	Harada [13]	39/M	(–)	(–)	Pyogenic spondylitis	Debridement	Alive
13	Suzuki [14]	65/F	Total gastrectomy for gastric cancer Cholecystectomy Splenectomy	(–)	Psoas abscess Epidural abscess	Drainage & Discectomy	Alive
14	Hayashi	87/M	cirrhosis	Diarrhea	Spontaneous bacterial peritonitis	Drainage	Alive

## 4 Conclusion

The results from the present case and the literature review suggest that *E. tarda* infection in the presence of an underlying disease such as hepatobiliary disease, malignancy, and/or diabetes mellitus has a poor prognosis. Although *E. tarda* infection is extremely rare, it is a life-threatening illness that can cause intestinal and extraintestinal infections. If necessary, early surgical intervention should be considered for cases of extraintestinal infection.

## Funding

This research did not receive any specific grant from funding agencies in the public, commercial, or not-for-profit sectors.

## Ethical approval

This report was reviewed and approved by the Institutional Review Board of JA GIFU Kouseiren Ibi Kosei Hospital.

## Consent

Informed consent was obtained from the patient for publication of this case report.

## Author contribution

H. Hayashi participated in the conception and design of the report.

Y. Murase, H. Sano, K. Nishio and I. Kumazawa reviewed and approved the final manuscript.

## Registration of research studies

N/A.

## Guarantor

The Guarantor is Hirokatsu Hayashi.

## Provenance and peer review

Not commissioned, externally peer-reviewed.

## Declaration of Competing Interest

The authors report no declarations of interest.

## References

[1] Janda J.M., Abbott S.L. (1993). Infections associated with the genus Edwardsiella: the role of Edwardsiella tarda in human disease. Clin. Infect. Dis..

[2] Matsushima S., Yajima S., Taguchi T., Takahashi A., Shiseki M., Totsuka K. (1996). A fulminating case of Edwardsiella tarda septicemia with necrotizing fasciitis. Kansenshogaku Zasshi.

[3] Tamura T., Ito Y., Tsuchiya R., Taguchi M., Terazawa A., Ishida S. (2009). A case of septic shock with necrotizing fasciitis caused by *Edwardsiella tarda*. J. Jpn. Soc. Intensive Care Med..

[4] Fujimoto M., Nakao K., Fujikawa K., Nishimura D. (2006). A case of rapidly progressive fatal septic shock following necrotizing fascilitis due to Edwardsiella tarda with hepatitis C virus related liver cirrhosis complicated hepatocellarcarcinoma. Kanzo.

[5] Sekine K., Suzuki T., Ukimura A. (2018). A case of sepsis due to Edwardsiella tarda with acute respiratory distress syndrome. JJHGM.

[6] Sugita N., Akamatsu J., Ueda K. (2015). A case of necrotizing fasciitis with alcoholic liver disease caused by Edwardsiella tarda. JSWC.

[7] Hara K., Ouchi H., Kitahara M., Shibano K., Miyauchi T., Ishiguro H. (2011). A case of fasciitis localized in the calf muscles associated with Edwardsiella tarda sepsis. Rinsho Shinkeigaku (Clin. Neurol.).

[8] Tokushige H., Izumiya C., Morita S., Matsushima S., Maeda N. (2015). A case of pelvic inflammatory disease caused by *Edwardsiella tarda*. Mod. Trends Obstet. Gynecol..

[9] Kobayashi F., Karasawa T., Yoshida T., Adachi W. (2019). A case of *Edwardsiella tarda* abscess of the uterine adnexa associated with appendiceal carcinoma. J. Jpn. Rural Med..

[10] Anno T., Kobayashi N. (2018). Infected subdural hematoma caused by *Edwardsiella tarda*. J. Rural Med..

[11] Ota T., Nakano Y., Nishi M., Matsuno S., Kawashima H., Nakagawa T. (2011). A case of liver abscess caused by Edwardsiella tarda. Intern. Med..

[12] Ohara Y., Kikuchi O., Goto T. (2012). Successful treatment of a patient with sepsis and liver abscess caused by Edwardsiella tarda. Intern. Med..

[13] Harada M., Yoshida H., Oomagari K., Sakai T., Abe H., Tanikawa K. (1990). A case of sepsis caused by Edwardsiella tarda complicated panophthalmitis and pyogenic spondylitis. Kansenshogaku Zasshi.

[14] Suzuki K., Yanai M., Hayashi Y., Otsuka H., Kato K., Soma M. (2018). Edwardsiella tarda bacteremia with Psoas and epidural abscess as a food-borne infection: a case report and literature review. Intern. Med..

[15] Agha R.A., Borrelli M.R., Farwana R., Koshy K., Fowler A., Orgill D.P., For the SCARE Group (2018). The SCARE 2018 statement: updating consensus Surgical CAse REport (SCARE) guidelines. Int. J. Surg..

[16] Garcia-Tsao G. (1992). Spontaneous bacterial peritonitis. Gastroenterol. Clin. N. Am..

[17] Blevrakis E., Anyfantakis D., Blevrakis E., Vlachakis I. (2016). Primary bacterial peritonitis in a previously healthy adolescent female: a case report. Int. J. Surg. Case Rep..

[18] Thalheimer U., Triantos C.K., Samonakis D.N., Patch D., Burroughs A.K. (2005). Infection, coagulation, and variceal bleeding in cirrhosis. Gut.

[19] Guarner C., Soriano G. (1997). Spontaneous bacterial peritonitis. Semin. Liver Dis..

[20] Onogawa T., Terayama T., Zen-yoji H., Amano Y., Suzuki K. (1976). Distribution of Edwardsiella tarda and hydrogen sulfide-producing Escherichia coli in healthy persons. Kansenshougaku Zasshi.

